# P-280. Risk Factors for Household Colonization with Extended-Spectrum Cephalosporin-Resistant Enterobacterales (ESCrE) in Botswana

**DOI:** 10.1093/ofid/ofae631.483

**Published:** 2025-01-29

**Authors:** Sukaina Shivji, Naledi Mannathoko, Mosepele Mosepele, Mosepele Mosepele, Robert Gross, Leigh Cressman, Anne Jaskowiak, Warren B Bilker, Kevin Alby, Laurel Glaser, Melissa Greenblatt, Laura Cowden, Alexa Patel, Kgotlaetsile Sewawa, Dimpho Otukile, Giacomo Paganotti, Margaret Mokomane, Evan Snitkin, Ebbing Lautenbach

**Affiliations:** Princeton University, Philadelphia, Pennsylvania; University of Botswana, Philadelphia, Pennsylvania; University of Botswana, Philadelphia, Pennsylvania; University of Botswana, Philadelphia, Pennsylvania; University of Pennsylvania, PHiladelphia, Pennsylvania; University of Pennsylvania Perelman School of Medicine, Philadelphia, Pennsylvania; University of Pennsylvania, PHiladelphia, Pennsylvania; University of Pennsylvania Perelman School of Medicine, Philadelphia, Pennsylvania; University of North Carolina, Chapel Hill, North Carolina; University of Pennsylvania, PHiladelphia, Pennsylvania; Hospital for Sick Kids, Philadelphia, Pennsylvania; University of Pennsylvania, PHiladelphia, Pennsylvania; University of Pennsylvania, PHiladelphia, Pennsylvania; Botswana-University of Pennsylvania Partnership, Gaborone, South-East, Botswana; University of Botswana, Philadelphia, Pennsylvania; University of Botswana, Philadelphia, Pennsylvania; University of Botswana, Philadelphia, Pennsylvania; University of Michigan, Ann Arbor, MI; University of Pennsylvania, PHiladelphia, Pennsylvania

## Abstract

**Background:**

The epidemiology of colonization with ESCrE in the community in low- and middle-income countries (LMICs) is largely uncharacterized. In the community, the household is of particular importance as it is the setting in which individuals spend a majority of their time. Identifying risk factors for household ESCrE colonization is critical to inform antibiotic resistance reduction strategies in LMICs.

**Figure:**
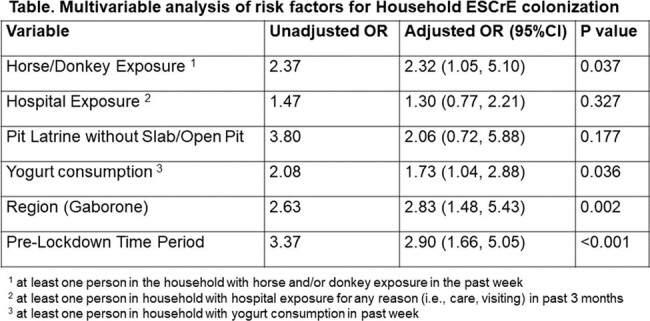

**Methods:**

Participants were enrolled from 1/1/20-9/4/20, with a pause 4/2/20-5/21/20 due to a country-wide COVID lockdown. This study was conducted in 6 clinics located in 3 regions in Botswana. In each clinic, we surveyed a random sample of outpatients presenting for care. We also invited each enrolled subject to refer household members. Only those subjects who referred at least one other household member were included. All participants had rectal swabs collected for identification of ESCrE. Data were collected on demographics, comorbidities, antibiotic use, healthcare exposures, travel, and farm/animal contact. Households were considered exposed if any member had the exposure of interest. Households with ESCrE colonization (cases) were compared to non-colonized households (controls). Bivariable and multivariable analyses were conducted to identify risk factors for household ESCrE colonization.

**Results:**

327 households were enrolled. The median (IQR) number of people enrolled per household was 3 (2-4) ranging from 2-10. Among enrolled households, the median proportion of household residents participating was 0.71 (0.5 – 1). The median (IQR) age of subjects was 18 years (5-34) and 304 (93%) households included at least one child. Of 327 households, 176 (54%) had at least one household member colonized with ESCrE. Independent risk factors for household colonization are noted in the table.

**Conclusion:**

ESCrE household colonization was common with evidence of geographic variability in ESCrE epidemiology as well as a possible role of animal exposure. The role of yogurt exposure requires additional study as we did not distinguish source (commercial, homemade). Further prospective studies of household ESCrE colonization with longitudinal assessments of exposures are required to identify effective prevention strategies.

**Disclosures:**

**Robert Gross, MD, MSCE**, Pfizer Inc: DSMB member for drug unrelated to study

